# Computational characterization of GRP78 binding sites on mitochondrial GPX4: implications for targeting ferroptosis in triple-negative breast cancer

**DOI:** 10.1038/s41598-025-33108-1

**Published:** 2026-01-10

**Authors:** Donia G. Youssef, Abdo A. Elfiky

**Affiliations:** 1 Department of Biophysics, Faculty of Sciences, Cairo University, Giza, 12613 Egypt; 2 School of Biotechnology, Badr University in Cairo, Badr City, Cairo, 11829 Egypt

**Keywords:** Triple-negative breast cancer, Endoplasmic reticulum stress, Lipid peroxidation-induced cell death, GRP78, GPX4, Structural bioinformatics, Ferroptosis modulation., Biochemistry, Cancer, Computational biology and bioinformatics, Drug discovery

## Abstract

About 20% of breast cancer cases are triple-negative breast cancer (TNBC), a highly aggressive subtype with limited therapeutic options. Emerging evidence suggests that ferroptosis — a form of regulated cell death — and stress-response pathways play critical roles in TNBC progression. We investigated the interaction between glucose-regulated protein 78 (GRP78), a central stress-response chaperone, and mitochondrial glutathione peroxidase 4 (mGPX4), a key regulator of ferroptosis resistance. Using a combined computational approach — including protein–protein docking, molecular dynamics (MD) simulations, and MM/GBSA free-energy calculations — we identified stable complexes between GRP78’s SBDβ domain and several regions of mGPX4. Docking with PRODIGY revealed binding affinities ranging from − 7.7 ± 0.5 to − 10.5 ± 0.6 kcal/mol, surpassing that of Pep42 (–6.9 ± 0.1 kcal/mol), with region III (the mitochondrial import sequence) showing the strongest binding (–10.5 ± 0.6 kcal/mol). HADDOCK scoring further highlighted region II as particularly favorable (–72.0 ± 5.4). After 100 ns of MD, MM/GBSA analysis estimated binding free energies from − 45.20 to − 86.39 kcal/mol, with the region-II complex exhibiting the highest affinity (–86.4 kcal/mol), driven predominantly by electrostatic and van der Waals interactions. This interaction could serve as a promising therapeutic target to undermine cancer cell survival by sensitizing TNBC cells to ferroptosis-inducing strategies.

**Supplementary Information:**

The online version contains supplementary material available at 10.1038/s41598-025-33108-1.

## Introduction

Breast cancer is the second most common cause of cancer-related death worldwide and the most common cancer in women. Human epidermal growth factor receptor 2 (HER2), progesterone receptors (PR), and estrogen receptors (ER) provide the basis for the classification^1,2^. Breast cancer is the second most common cause of cancer-related death worldwide and the most common cancer in women. Human epidermal growth factor receptor 2 (HER2), progesterone receptors (PR), and estrogen receptors (ER) provide the basis for the classification^1^. Triple-negative breast cancer (TNBC), which is defined by the lack of ER, PR, and HER2 expression, accounts for about 20% of cases. One of the most aggressive forms of breast cancer, TNBC, frequently results in poor outcomes since it spreads to other organs such as the brain, bone, and lungs^3^. Since TNBC lacks the common hormone receptors and HER2 target, and its molecular drivers remain poorly defined, reliable diagnostic and prognostic markers are still lacking. Consequently, chemotherapy has been the mainstay of treatment for TNBC for decades. Although TNBC often responds to chemotherapy, it has a lower overall survival rate, a higher chance of distant relapse, and a higher possibility for metastases compared with other breast cancer subtypes^4^.

The unfolded protein response (UPR) can be triggered by endoplasmic reticulum (ER) stress caused by hypoxia, altered glucose metabolism, and insufficient perfusion in tumours. Known as HSPA5 (heat-shock protein A5), glucose-regulated protein 78 (GRP78) is a 70-kDa heat-shock protein that serves as a molecular chaperone and an essential regulator of the unfolded protein response (UPR)^5^. It is overexpressed in many cancers, where it supports tumor survival, proliferation, chemoresistance, angiogenesis, and metastasis. While GRP78 is best known for binding misfolded proteins in the ER, it also appears on the surface of tumor cells. In regard to its structure, GRP78 has a C-terminal substrate-binding domain (SBD) that recognizes unfolded polypeptides and prevents their aggregation, as well as an N-terminal nucleotide-binding domain (NBD) that hydrolyzes ATP^6,7^. When the endoplasmic reticulum (ER) is under stress, GRP78 dissociates from the three unfolded protein response (UPR) sensors: activating transcription factor 6 (ATF6), protein kinase RNA-like endoplasmic reticulum kinase (PERK), and inositol-requiring enzyme 1 (IRE1). This mechanism assists in the restoration of balance by enhancing the ability of proteins to fold and decreasing the creation of new proteins^8^.

Numerous investigations have shown that by maintaining the stability of GPX4, GRP78 also helps cancer cells survive ferroptosis. Ferroptosis is a recently identified type of controlled cell death (RCD) caused by lipid hydroperoxide buildup that is dependent on iron. Biochemically, this process results from the accumulation of reactive oxygen species (ROS) produced from lipids due to the loss of function of the lipid repair enzyme glutathione peroxidase 4 (GPX4). Consequently, cancer cells, particularly those found in TNBC, are made possible by this interaction to evade ferroptosis-inducing agents and endure oxidative stress^9,10^. This highlights the significant role of GRP78 in tumor progression and resistance to treatment^1,11^.

Computational approaches have become essential in contemporary drug discovery, reducing the time, expense, and manual effort required by traditional laboratory methods. In this study, we combine three primary in silico techniques—molecular docking, molecular dynamics (MD) simulations, and binding free-energy calculations—to predict and quantify protein–protein binding affinities. Merging computational biophysics with bioinformatics further accelerates our insight into disease processes: pipelines that integrate predictive modeling with docking and MD have successfully revealed how particular mutations influence protein stability and interaction profiles^12,13^. At the same time, advances in structural prediction—most notably AlphaFold2—have greatly improved our ability to generate high-accuracy protein models, enabling reliable docking and interaction analyses even when experimental structures are lacking^14^. By uniting precise structural modeling, flexible docking, dynamic simulation, and free-energy evaluation, we can map key contacts and conserved regions at protein interfaces. This interdisciplinary framework not only enriches our molecular understanding but also bolsters the discovery of novel therapeutic targets. Here, we apply these integrated computational methods to dissect the GRP78–GPX4 interaction, aiming to clarify how this complex may promote TNBC cell survival and inhibit ferroptotic death.

## Materials and methods

### 3D structure prediction

The UniProt database provided the FASTA format sequence of the 197 amino acid mitochondrial GPX4 (mGPX4) protein^15^. Mitochondrial GPX4 differs from the cytosolic form by a 27-amino-acid N-terminal extension that directs its import into mitochondria^16^. The 3D structure of mGPX4 was generated using AlphaFold2 (version 1.5.5)^17^. The GalaxyRefine server refined the model, and Molprobity was used to validate it^18,19^. We chose four GPX4 regions— region I (R1) (C7–C16), region II (R2) (C16–C29), region III (R3) (C7–C29) and region VII (R7) (C93–C102)—to test whether GRP78 can bind the mitochondrial targeting regions region I – region III. Each region was pairwise-aligned with the Pep42 peptide using EMBL-EBI’s Clustal Omega (default settings). Notably, Pep42’s cyclic conformation is the key feature driving its selective recognition by GRP78^20^. The approach enabled selective delivery of doxorubicin to tumor cells exhibiting upregulated cell-surface GRP78, thereby improving target specificity and minimizing off-target toxicity^21^. Important physicochemical characteristics, such as the fraction of nonpolar residues and the grand average of hydropathy (GRAVY), were analyzed in order to compare areas to Pep42. The ProtParam tool on the ExPASy site and the EMBOSS Pepstats service at EMBL-EBI were used for these investigations, respectively^20,22^.

The Protein Data Bank (PDB) provided the GRP78 x-ray diffraction structure, which was identified by PDB ID: 6ASY and had a resolution of 1.85 Å. To obtain the structure prepared to undergo docking, we applied the PyMOL software. Only one protein chain remained in the PDB file after water molecules, ions, ligands, and other chains were eliminated^23^. To ensure compatibility with downstream analyses and avoid residue ID conflicts, 1000 was added to each residue number in the GRP78 structure after preparation.

### Full-length mGPX4 envelope - GRP78 binding

Protein-protein docking studies were conducted using the HADDOCK 2.4 online service to examine the binding interactions between the GRP78 protein (PDB ID: 6ASY) and the full-length mGPX4 envelope protein^24^. Additionally, GRP78’s docking with the Pep42 peptide served as a reference model. Active residues in certain areas of mGPX4 and GRP78 SBDβ have already been identified^24^. ILE426, THR428, VAL429, VAL432, THR434, PHE451, SER452, VAL457, and ILE459 are the GRP78 residues implicated according to previous work^25^. The full sequence of the cyclic peptide Pep42 and particular GPX4 segments, such as region I (C7-C16), region II (C16-C29), region III (C7-C29), and region VII (C93-C102), were regarded as active residues. Both binding scores and PRODIGY-predicted binding energies were computed for every resultant complex^26,27^. To ascertain the quantity and kinds of interactions between complexes, each complex was also examined using the Protein–Ligand Interaction Profiler (PLIP) website^28^. For visualization and docking analysis, PyMOL and Chimera X were utilized^23,29^.

### System Preparation and molecular dynamics workflow for GRP78 binding to Full-Length mGPX4

The conformational stability and interaction kinetics between GRP78 and full-length mGPX4 were examined using molecular dynamics (MD) simulations. The Protein Data Bank provided the crystal structure of GRP78 (PDB ID: 6ASY), which was then used in protein–protein docking with mGPX4 to determine the most favourable binding orientations. The top-scoring complexes (lowest predicted ΔG) for simulation were selected for further analysis. In addition to separate MD runs on each protein. Every system underwent a standard MD workflow—energy minimization, equilibration, and production runs—to evaluate binding stability, structural behavior, and interaction energetics.

In GROMACS 2023.3, MD simulations were conducted in accordance with a classical, unbiased protocol that lasted 100 ns^30^. Initial system files were built with the Solution Builder module of the CHARMM-GUI web server^31^. In each arrangement, the matching PDB structure was positioned inside a rectangular TIP3P water box with a 1.5 nm margin on all sides. To neutralize the system and reach a physiological salt concentration of 154 mM, sodium and chloride ions were added. All simulations employed the CHARMM36m force field.

After solvation and ion placement, energy minimization was conducted until the maximum atomic force fell below 1,000 kJ·mol⁻¹·nm⁻¹. The systems were subsequently equilibrated in the NVT ensemble for a duration of 100 ps at a temperature of 310 K, employing the V-rescale thermostat for this process. Production runs were conducted for 100 ns within the NPT ensemble at a temperature of 310 K and a pressure of 1 bar, regulated by the V-rescale thermostat and the Parrinello–Rahman barostat. Hydrogen-involving bonds were constrained using LINCS, while long-range electrostatics were addressed through Particle Mesh Ewald with a cutoff of 1.2 nm. A leap-frog integrator was employed, using a 1 fs timestep during equilibration and 2 fs during production. Trajectory frames were recorded every 0.1 ns (1,000 frames total). Periodic boundary artifacts were removed. Custom scripts in the VMD Tk console were utilized to calculate various structural and dynamic metrics, including root-mean-square deviation (RMSD), root-mean-square fluctuation (RMSF), radius of gyration (Rg), intermolecular hydrogen-bond counts, and solvent-accessible surface area (SASA)^32^.

As controls, we ran separate 100-ns MD simulations of unbound GRP78 and of full-length mGPX4 using the same conditions. These baseline trajectories serve as references for the proteins’ intrinsic dynamics, allowing direct comparison with the GRP78–mGPX4 complexes to evaluate their relative stability.

#### MM-GBSA binding free energy calculation

The trajectory from the molecular dynamics simulation was used to compute the binding free energy (Gibbs free energy) using MM-GBSA calculations using the gmxMMPBSA tool. Van der Waals forces, electrostatic energies, solvation energies, and entropy corrections are all calculated. Additionally included are energy decompositions with bond, angle, and dihedral components. Energy contributions for each compound were calculated using conformational snapshots taken from the trajectory over a predetermined period of time. The Poisson-Boltzmann equation was used to compute the electrostatic solvation, whereas the solvent-accessible surface area was used to get the non-polar solvation energy^33,34^.

## Results and discussion

### *Comparative 3D structural analysis of Pep42 and functional regions of GPX4*

The mGPX4 3D structure was generated with AlphaFold2 (v1.5.5) via the ColabFold pipeline. By coupling accelerated MSA searches with AlphaFold2 or RoseTTAFold, ColabFold delivers a 40–60× speedup in structure and complex predictions without sacrificing accuracy. The resulting mGPX4 model achieved a mean pLDDT of 89.4, signifying a well-resolved global topology (pLDDT ≥ 85 denotes high confidence)^17^.

We evaluated model confidence by examining per-residue pLDDT scores across the full mGPX4 sequence. The catalytic core—including Region 7, which was selected for docking—exhibited high pLDDT values (> 85) and clearly defined secondary and tertiary structure. In contrast, the N-terminal mitochondrial targeting segment (residues 1–27) scored below 50, reflecting its intrinsic disorder and lack of a stable fold prior to import. Despite this, we retained the unstructured N-terminus for docking because GRP78 (HSPA5) naturally binds unfolded or partially folded N-terminal motifs of its client proteins, particularly under stress. Modeling GRP78 interactions with this flexible region, therefore, captures a biologically relevant scenario for probing mGPX4 chaperone engagement.

To avoid relying solely on the disordered N-terminal segment, we also carried out docking on Region 7, a well-defined, high-confidence structural element of GPX4. By pairing the biologically relevant N-terminal fragment with this robust structured region, our dual-region approach offers a balanced, comprehensive evaluation of potential GRP78–mGPX4 interaction sites.

We refined the mGPX4 model with GalaxyRefine and then assessed it using MolProbity. The final structure exhibited excellent overall stereochemistry. Although 5.64% of the Ramachandran outliers clustered in the intrinsically disordered N-terminal region, both the well-folded catalytic core and Region 7 showed very few deviations and high stereochemical quality, confirming their suitability for docking (see Table 1).

**Table 1:** Molprobity analysis of the refined mGPX4 structure.

Validation Metrics	Refined mGPX4 Model
Clash score	3.88
Poor rotamers Goal: <0.3%	1.23%
Favored rotamers. Goal: >98%	156 (95.71%)
Ramachandran outliers Goal: <0.05%	5.64%
Ramachandran favored Goal: >98%	165 (84.62%)
Cβ deviations > 0.25Å Goal:0	2.22%
Residues with Bad Bonds Goal:0%	0%
Residues with Bad Angles Goal: <0.1%	20 (0.94%)
MolProbity score	1.91

**Fig. 1:**
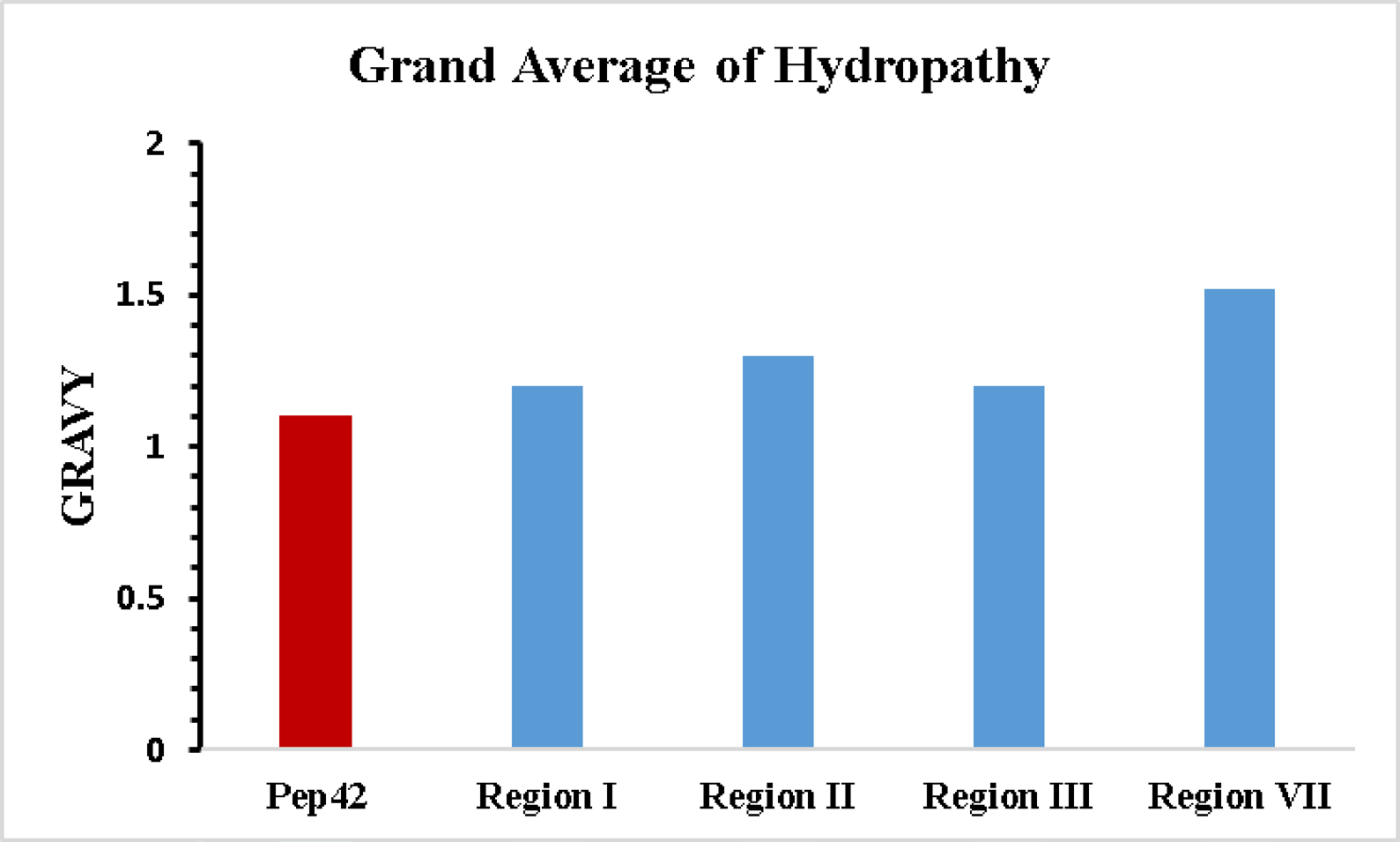
Comparison of the grand average of hydropathy (GRAVY) index values for the four selected regions (region I (R1) (C7–C16), region II (R2) (C16–C29), region III (R3) (C7–C29) and region VII (R7) (C93–C102)) of the mitochondrial GPX4 (mGPX4) (blue) with that of the Pep42 peptide (red).

Sequence alignment revealed identities of 30%, 30.77%, 38.46%, and 42.86% between GPX4 regions R1, R2, R3, and R7, respectively, and Pep42. Notably, several conserved residues were observed: in R1, C7, L9, L10, P12, and C16 correspond to C1, V3, A4, P6, and C13 in Pep42; in R2, L19, A20, A21, P22, G23, M28, and C29 align with V3, A4, L5, P6, G7, V12, and C13; and in R3, C7, L19, A20, A21, P22, G23, M28, and C29 match C1, V3, A4, L5, P6, G7, V12, and C13. Similarly, R7 shares C93, L95, A99, and P101 with C1, V3, A4, and P6 in Pep42. These conserved cysteine and proline residues, along with hydrophobic positions, suggest structural compatibility. Furthermore, GPX4-derived peptides adopt a cyclic conformation via a disulfide bond between terminal cysteines, reinforcing potential functional similarity beyond sequence identity. GPX4 regions I, II, III, and VII exhibit GRAVY scores of 1.2, 1.3, 1.2, and 1.5, respectively, whereas Pep42 records a value of 1.1, indicating comparable overall hydrophobicity (Fig. 1). This resemblance is biologically significant because Pep42 engages GRP78 predominantly through hydrophobic contacts, implying that GPX4 fragments with similar hydrophobic profiles could exploit the same binding mechanism. As a molecular chaperone, GRP78 detects and binds exposed hydrophobic patches on client proteins, thereby promoting their correct folding and stabilization. Consequently, the hydrophobic stretches within GPX4 offer a plausible explanation for its interaction with GRP78, potentially enhancing mitochondrial GPX4 recruitment or stabilization. Additionally, approximately 85% of Pep42 and about 80%, 93%, 87% and 90% of the four GPX4 segments consist of nonpolar residues, further reinforcing the likelihood of shared physicochemical properties that facilitate analogous binding behavior.

### ***Full-length mGPX4 envelope - GRP78 binding***

**Fig. 2:**
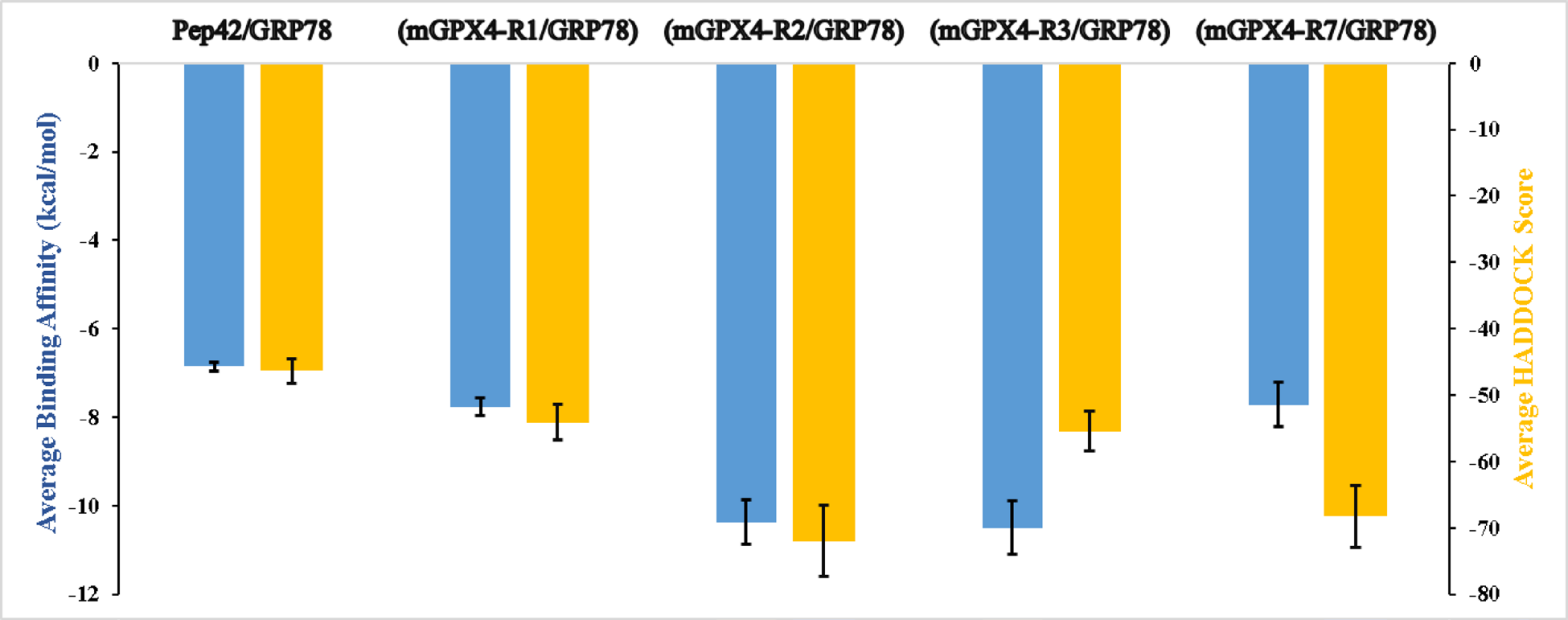
*Binding affinity predictions for the four GRP78–mGPX4 complexes. The PRODIGY-predicted binding affinities (in kcal/mol) are shown as blue bars*,* while the corresponding HADDOCK scores are represented as orange bars. Lower values indicate stronger binding and better docking quality.*

The docking trials revealed binding modes, highlighting the best-formed complexes of GRP78 and mGPX4. Figure 2 illustrates the predicted alignment of GRP78’s SBDβ with full-length mGPX4, highlighting the binding affinities (ΔG, kcal/mol) estimated by PRODIGY with error bars denoting the standard deviations, which varied from − 7.7 ± 0.5 to −10.5 ± 0.6 kcal/mol—exceeding the affinity of the cyclic peptide Pep42 (−6.9 ± 0.1 kcal/mol). Region III of the mGPX4, located within the mitochondrial import region, exhibited the highest binding affinity, characterized by an average ΔG of −10.5 ± 0.6 kcal/mol. Other regions exhibited marginally lower affinities: −7.8 ± 0.2 kcal/mol for region I, −10.4 ± 0.5 kcal/mol for region II, and − 7.7 ± 0.5 kcal/mol for region VII.

Based on HADDOCK docking results, Region II stands out as the preferred binding site, with a score of − 72.0 ± 5.4—considerably better than region I (–54.1 ± 2.6), region III (–55.5 ± 3.0), region VII (–68.3 ± 4.6), and the Pep42 reference (–46.4 ± 1.8). Structurally, region II offers a highly complementary interface for GRP78’s SBDβ: it adopts a stable helix–loop conformation rich in solvent-exposed hydrophobic and polar side chains. This arrangement nestles perfectly into GRP78’s hydrophobic groove while simultaneously forming a network of stabilizing hydrogen bonds. Moreover, the relatively unobstructed surface of region II creates a continuous interaction patch that buries more surface area than the other regions. These combined features explain why, even though its predicted ΔG is on par with region III, region II delivers the most favorable docking energetics and thus represents the most biochemically credible binding site (see the supplementary Table S1).

#### Mechanistic insights into GRP78 SBDβ–mGPX4 interaction

The interaction pattern of the highest-ranked docking clusters was examined using PLIP software and is presented in Table 2. The primary modes of interaction between mGPX4 and the SBDβ of GRP78 include hydrogen bonds and hydrophobic interactions. Furthermore, π-stacking was observed in the GRP78–cyclic Pep42 peptide complex, while salt bridges were detected in the mGPX4-R3/GRP78and mGPX4-R7/GRP78complexes.

**Fig. 3:**
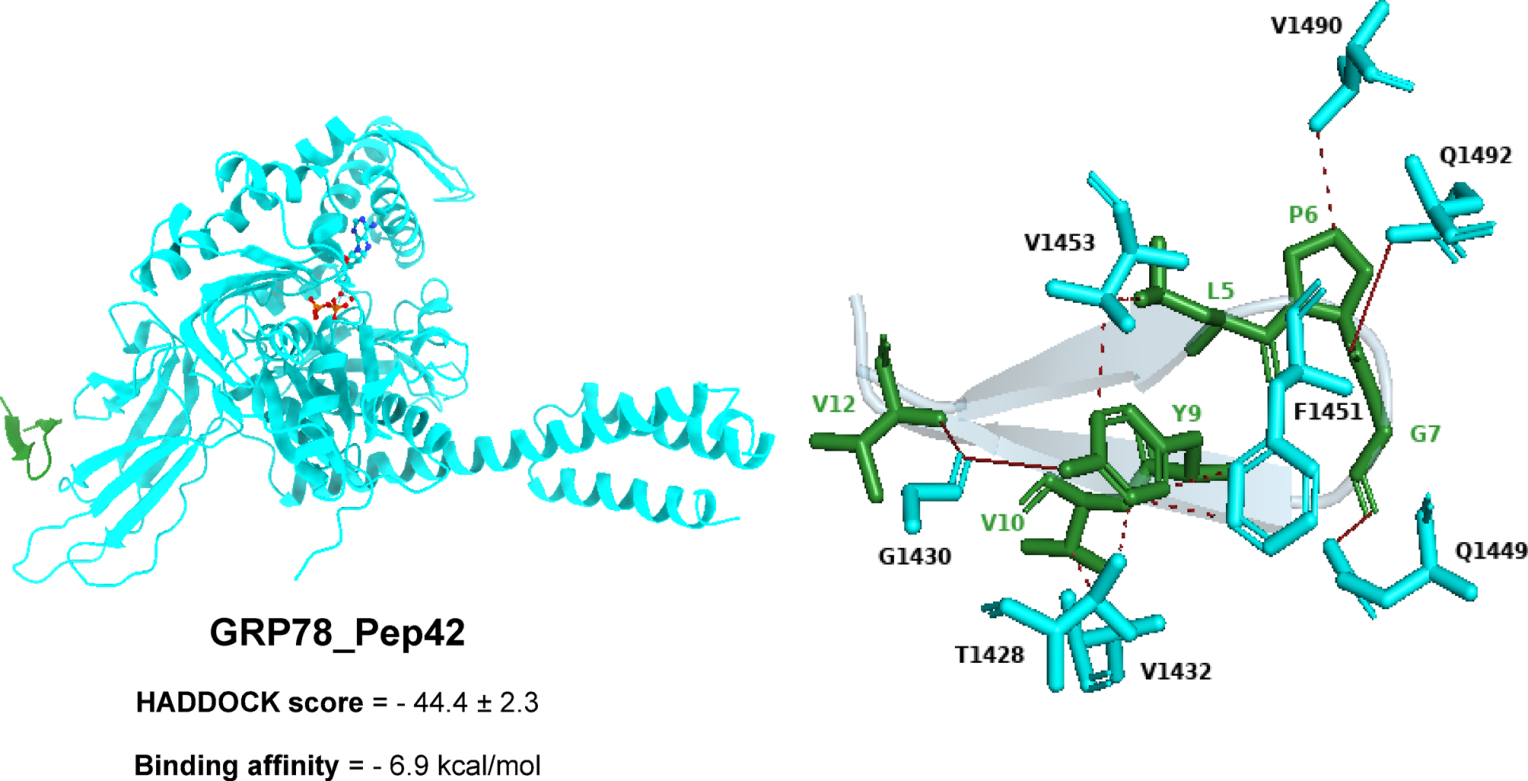
Three-dimensional representations depict the binding modes and molecular interactions of the cyclic Pep42 peptide within the active site of the GRP78 SBDβ domain.

The SBDβ domain of GRP78, consisting of residues G1430(2), Q1449, and Q1492, establishes four hydrogen bonds with the V12, Y9, G7, and P6 residues of the cyclic Pep42 peptide. Additionally, there are seven hydrophobic contacts involving T1428, V1432, F1451(2), V1453(2), and V1490 with the Y9(4), V10, L5, and P6 residues of the Pep42 peptide. Moreover, π-stacking interactions occur between F1451 and Y9(6) (Fig. 3).

**Table 2:** Interaction pattern analysis between the GRP78 SBDβ domain (PDB ID: 6ASY) and the four mGPX4 regions. (a) π–π stacking interactions; (b) salt bridges. Active residues of the GRP78 SBDβ domain are highlighted in bold. The GRP78–Pep42 Cyclic peptide complex was used as a reference model.

Top-ranked cluster	H-bonding			Hydrophobic interaction			Other interactions		
	*N*	GRP78 Residues	Pep42/GPX4 Residues	*N*	GRP78 Residues	Pep42/GPX4 Residues	*N*	GRP78 Residues	Pep42/GPX4 Residues
**Pep42/GRP78**	4	G1430(2), Q1449, Q1492	V12, Y9, G7, P6	7	**T1428**, **V1432**, **F1451(2)**, V1453(2), V1490	Y9(4), V10, L5, P6	**1**	F1451	Y9(6) ^**(a)**^
**Complex1** **(mGPX4-R1/GRP78)**	3	**T1434**, **S1452**, R1466	L10, A13, S2	4	**T1434**, P1467, L1468, V1490	L10, L3, L6, L14			
**Complex2** **(mGPX4-R2/GRP78)**	8	K1447, Q1449 (2), **S1452(2)**, V1453, A1486, G1489	G17, A21(2), G25, T27(2), C29(2)	3	**T1434**, **F1451**, V1453	A21, L24(2)			
**Complex3** **(mGPX4-R3/GRP78)**	8	**T1434(2)**, Q1449, I14450, P1487, R1488, Q1492, K1512	A18(2), L19, A21, C29, T27, L24, G26	4	**V1432**, **T1434**, P1467, L1468	A18(2), A13(2)	1	D1333	R5(3) ^**(b)**^
**Complex4** **(mGPX4-R7/GRP78)**	4	**V1432**, **S1452(2)**, V1453	E92, R96(2), K58	5	**V1429**, **V1432**, P1485(2), R1488	K126, E92, V54, H52, D57	1	R1488	D57 (2) ^**(b)**^

**Fig. 4:**
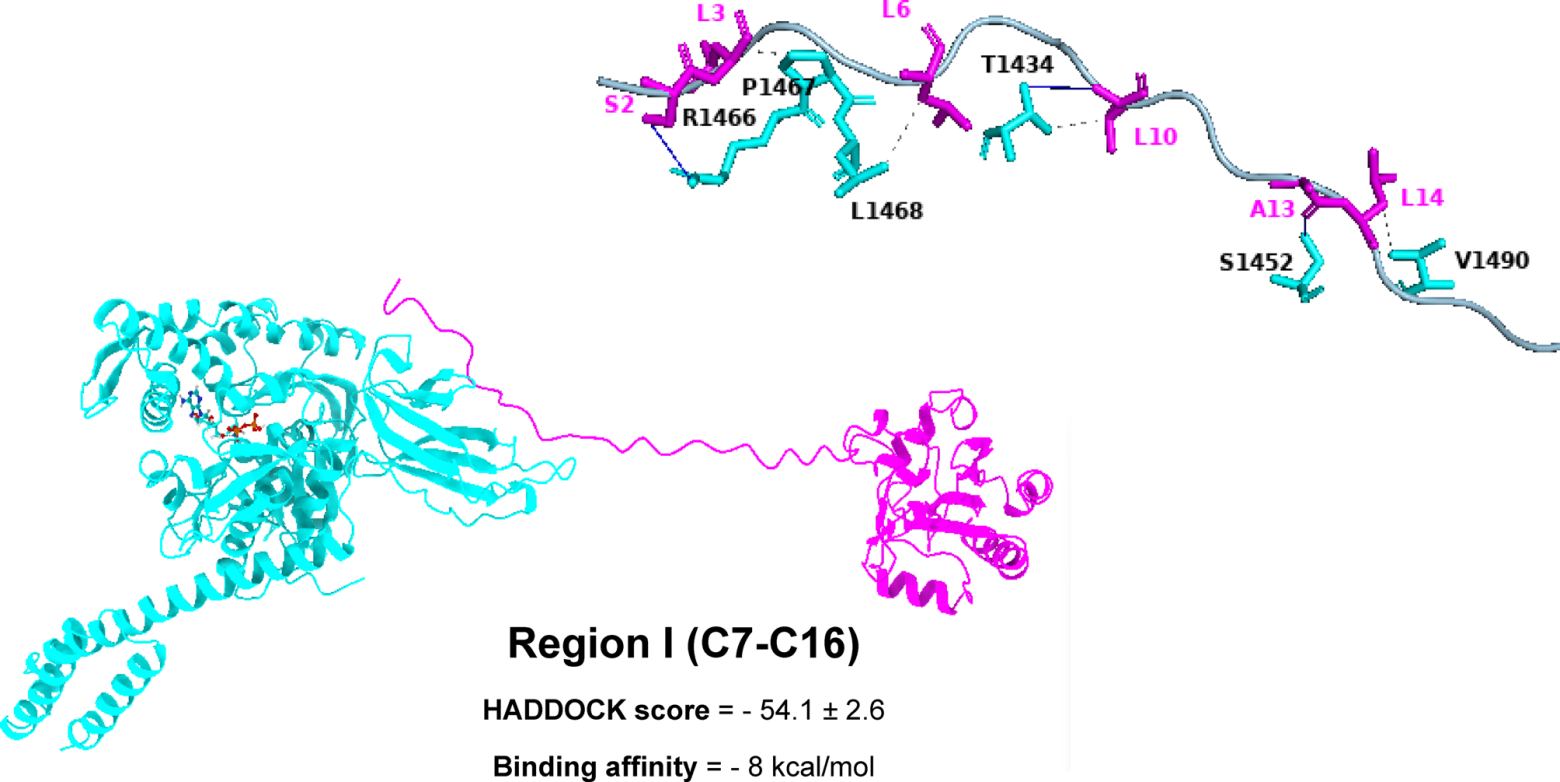
3D models showing the molecular interactions and binding mechanisms of mGPX4 Region I at the GRP78 SBDβ domain’s active region.

**Fig. 5:**
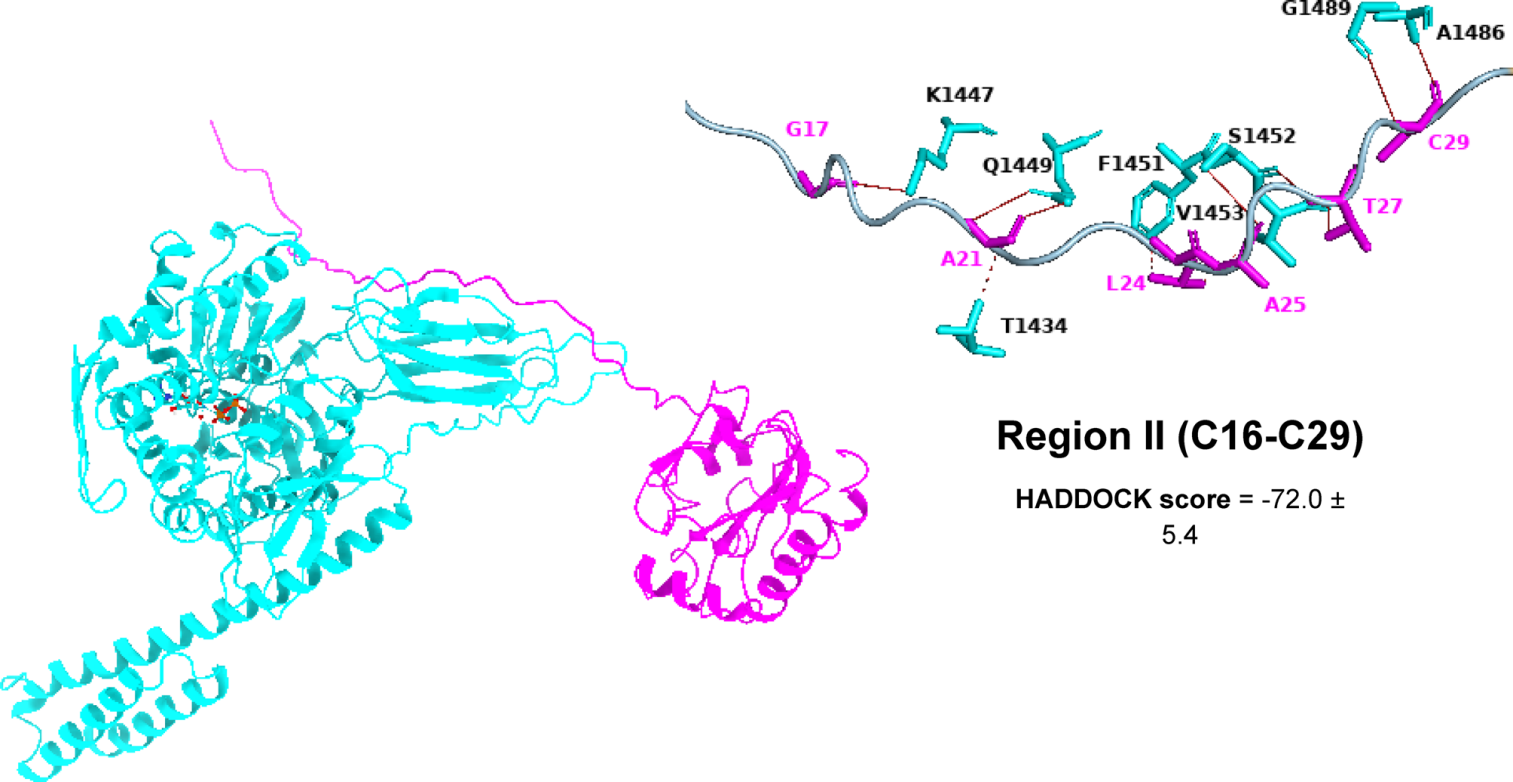
3D models showing the molecular interactions and binding mechanisms of mGPX4 Region II at the GRP78 SBDβ domain’s active region.

**Fig. 6:**
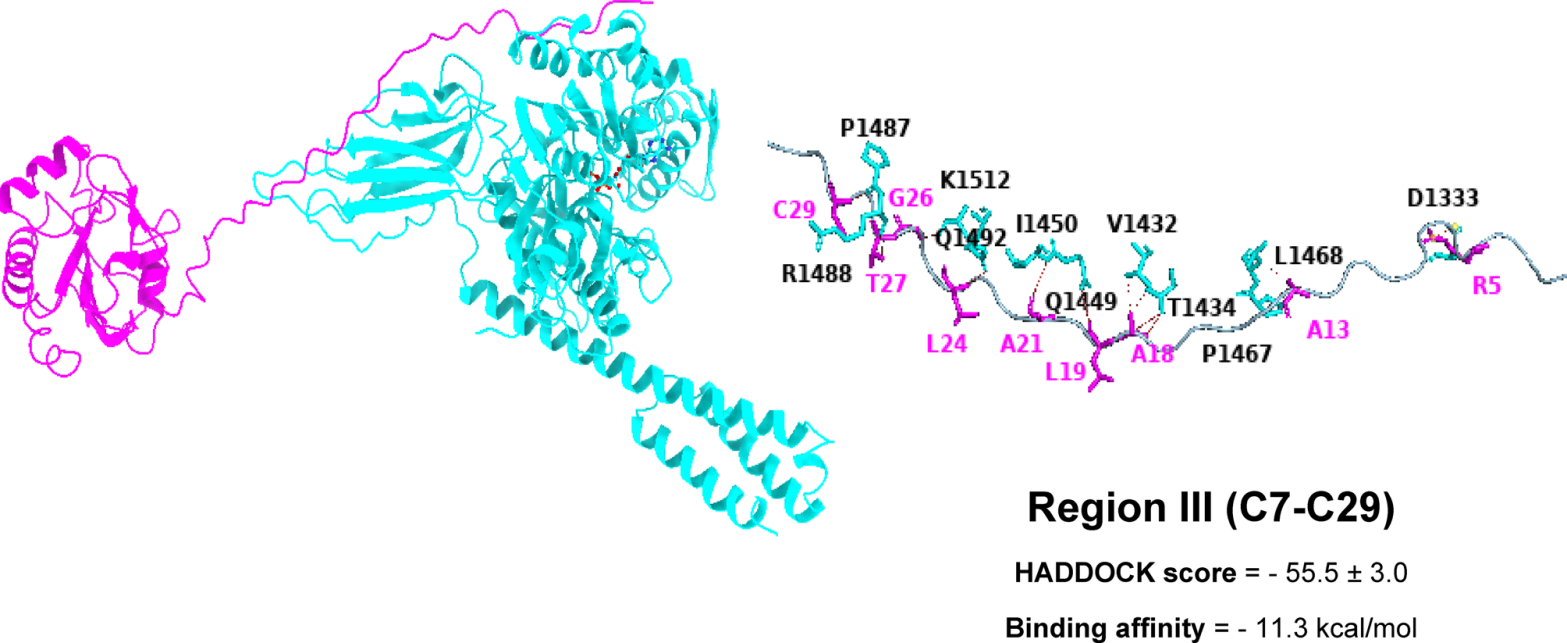
3D models showing the molecular interactions and binding mechanisms of mGPX4 Region III at the GRP78 SBDβ domain’s active region.

**Fig. 7:**
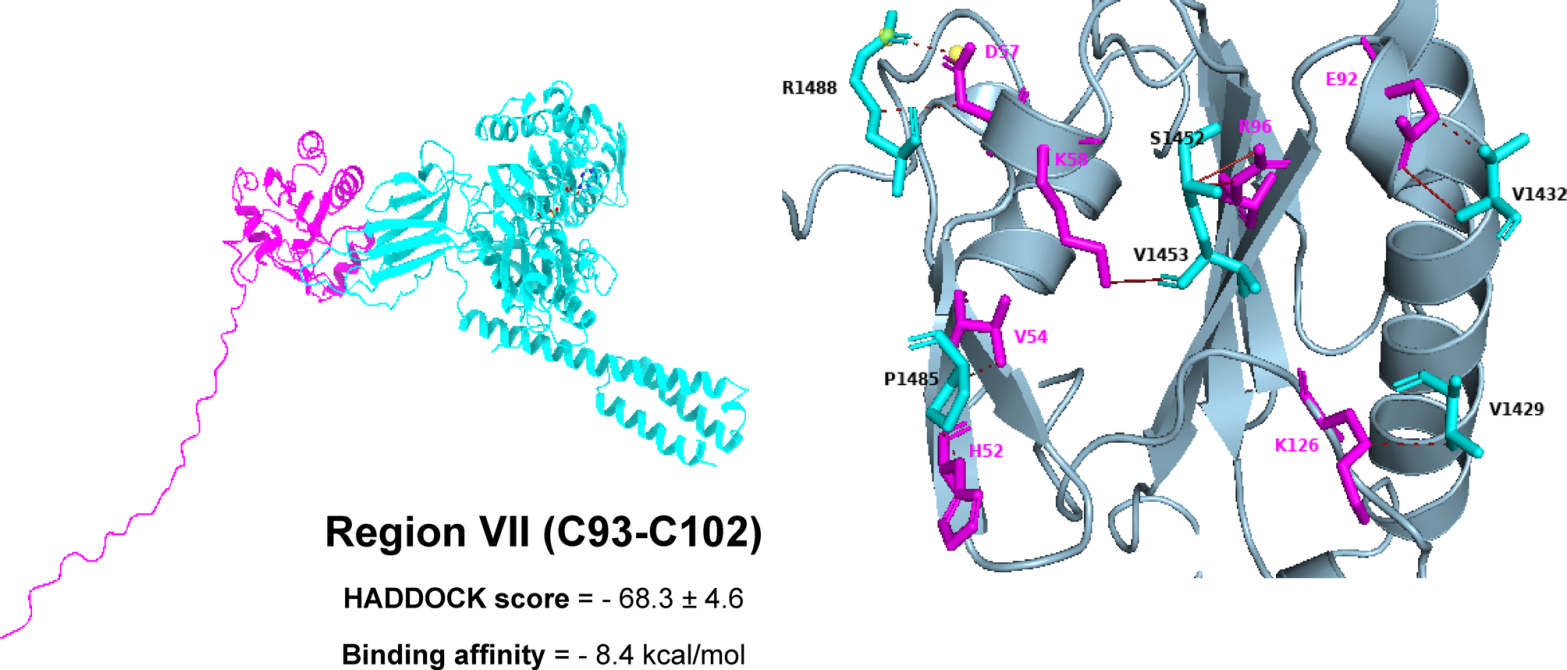
3D models showing the molecular interactions and binding mechanisms of mGPX4 Region VII at the GRP78 SBDβ domain’s active region.

The mGPX4 envelope displays a potential binding configuration as depicted in Figs. 4, 5, 6 and 7. The SBDβ domain of GRP78 forms three hydrogen bonds with residues T1434, S1452, and R1466, interacting with L10, A13, and S2 of region I (C7-C16). These interactions position the N-terminal loop of mGPX4 into the shallow entrance of the GRP78 binding groove. Additionally, it establishes four hydrophobic interactions involving T1434, P1467, L1468, and V1490 with L10, L3, L6, and L14 of region I, effectively creating a hydrophobic cradle that locks the N-terminal segment in place. This dual polar–nonpolar complementarity appears to stabilize the initial docking orientation of the peptide (Fig. 4).

In region II (C16-C29), GRP78 SBDβ establishes eight hydrogen bonds and three hydrophobic interactions, involving residues K1447, Q1449 (2), S1452(2), V1453, A1486, and G1489, forming H-bonds with G17, A21(2), G25, T27(2), and C29(2). These bonds create a continuous chain of polar contacts that align the central segment of mGPX4 along the β-sheet edge of GRP78. Additionally, hydrophobic contacts with T1434, F1451, and V1453 interact with A21 and L24(2), forming a hydrophobic spine that further stabilizes this central anchoring region. Mechanistically, this extensive polar–hydrophobic interplay likely constitutes the core stabilizing interface for mGPX4 recognition (Fig. 5).

In region III (C7-C29), eight stable hydrogen bonds are formed by GRP78 SBDβ with T1434(2), Q1449, I14450, P1487, R1488, Q1492, and K1512 interacting with A18(2), L19, A21, C29, T27, L24, and G26, along with four hydrophobic interactions involving V1432, T1434, P1467, and L1468 with A18(2), and A13(2). These interactions form a bipartite anchoring system, in which the β-strand interactions mediate alignment while hydrophobic residues clamp the peptide laterally. A salt bridge between D333 of GRP78 and R5 of mGPX4 further reinforces electrostatic complementarity, likely contributing to specificity by stabilizing the N-terminal charge distribution (Fig. 6).

In region VII (C93-C102), GRP78 SBDβ forms four hydrogen bonds with V1432, S1452(2), and V1453 interacting with E92, R96(2), and K58 alongside five hydrophobic contacts with V1429, V1432, P1485(2), and R1488 interacting with K126, E92, V54, H52, and D57. These interactions appear to create a secondary stabilizing interface, possibly capturing the C-terminal helix of mGPX4 against the lateral wall of the SBDβ. The salt bridge between R488 and D57 provides an additional long-range electrostatic lock, which may prevent dissociation and contribute to the prolonged chaperone–substrate engagement characteristic of GRP78 (Fig. 7).

In combination, these binding modes show that GRP78 secures mGPX4 by aligning β-strands, clamping hydrophobic patches, and deploying targeted electrostatic anchors—together creating an integrated mechanism for substrate recognition and stabilization.

###  Conformational dynamics and energetic characterization of GRP78 binding to mGPX4

Understanding the conformational dynamics of mGPX4 and the effect of GRP78 binding is essential for elucidating how this chaperone protein modulates mGPX4 stability and function. The study employs molecular dynamics simulations to analyze mGPX4 both on its own and in complex with GRP78 at various sites—the mitochondrial import regions (Regions I, II, and III) as well as the distal Region VII. We benchmarked the stability of each GRP78–mGPX4 complex against control simulations of GRP78 and mGPX4 in their unbound forms. This comparison ensured that any changes in RMSD, RMSF, Rg, or SASA could be ascribed to GRP78 binding, rather than to the proteins’ intrinsic flexibility.

**Fig. 8:**
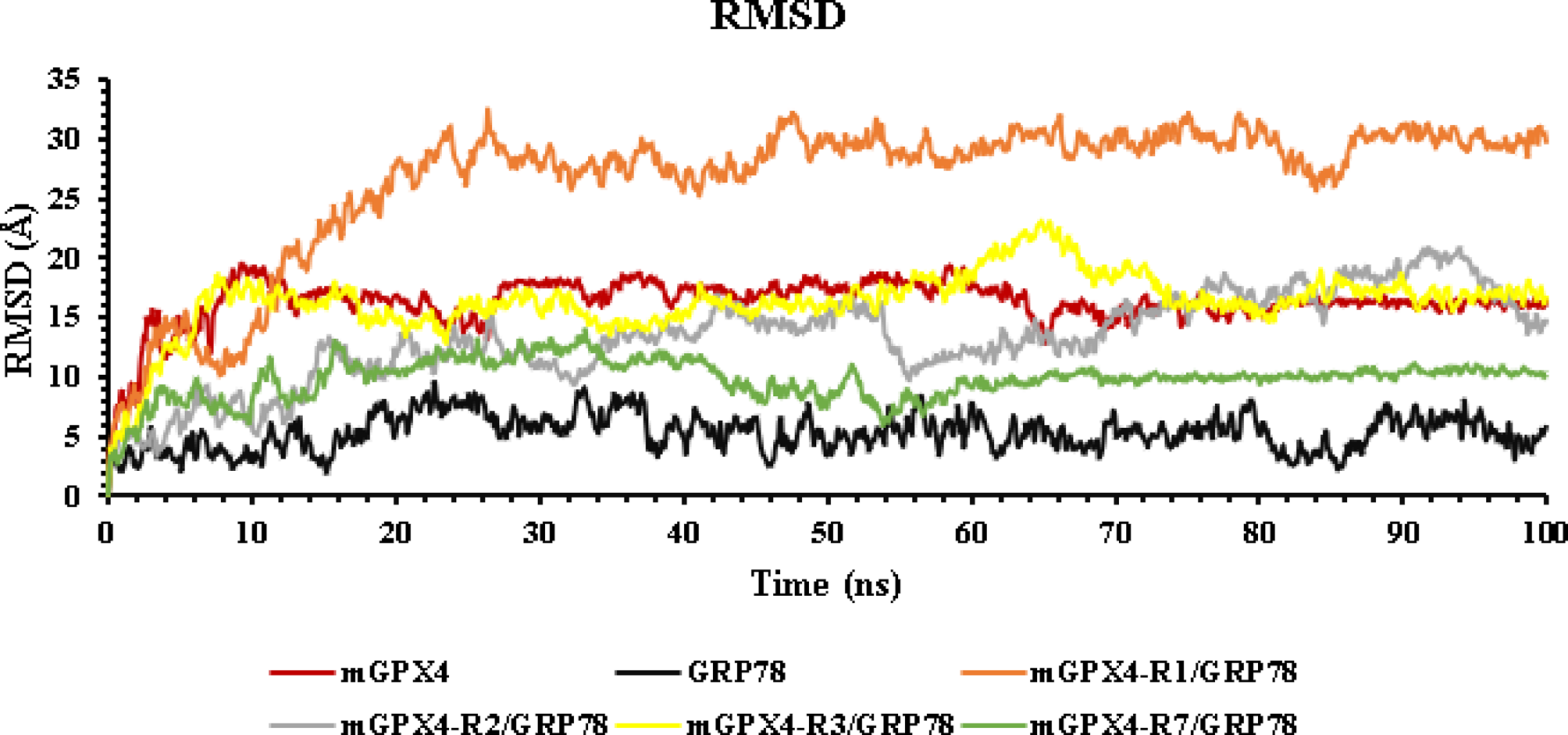
Root-mean-square deviation (RMSD) for each protein and the 4 complexes (GRP78-mGPX4). mGPX4 (red), GRP78 (black), complex1(mGPX4-R1/GRP78) (orange), complex2 (mGPX4-R2/GRP78) (gray), complex3 (mGPX4-R3/GRP78) (yellow), and complex4 (mGPX4-R7/GRP78) (green).

The study of RMSD, RMSF, Rg, SASA, and hydrogen bonds shows that GRP78 has a region-specific impact on the conformational stability of mGPX4. To assess the overall conformational stability of mGPX4 and its complexes with GRP78, the RMSD of backbone atoms over 100 ns was analyzed (Fig. 8). RMSD shows considerable fluctuations, with an average RMSD of 16.31 Å and peaks up to 19.48 Å. In contrast, the isolated GRP78 exhibited significant stability (average RMSD 5.27 Å), according to its secondary structure, which is primarily made up of beta-sheets and alpha-helices with a small number of loop regions. GPX4, which is a small protein characterized by a higher number of loops, a structural feature that contributes to the protein’s increased dynamic flexibility.

The GRP78–mGPX4 complexes involving regions VII and II showed the largest decreases in mGPX4 RMSD, averaging 9.83 Å and 13.34 Å, respectively, reflecting increased structural stability upon GRP78 association. These findings are in line with previous reports suggesting that GRP78 protects target proteins from misfolding and degradation^9,10^. Conversely, complexes formed at regions I and III exhibited higher average RMSDs (26.53 Å and 28.12 Å), suggesting that GRP78 binding at these positions may be linked to modulating mitochondrial import under stress.

**Fig. 9:**
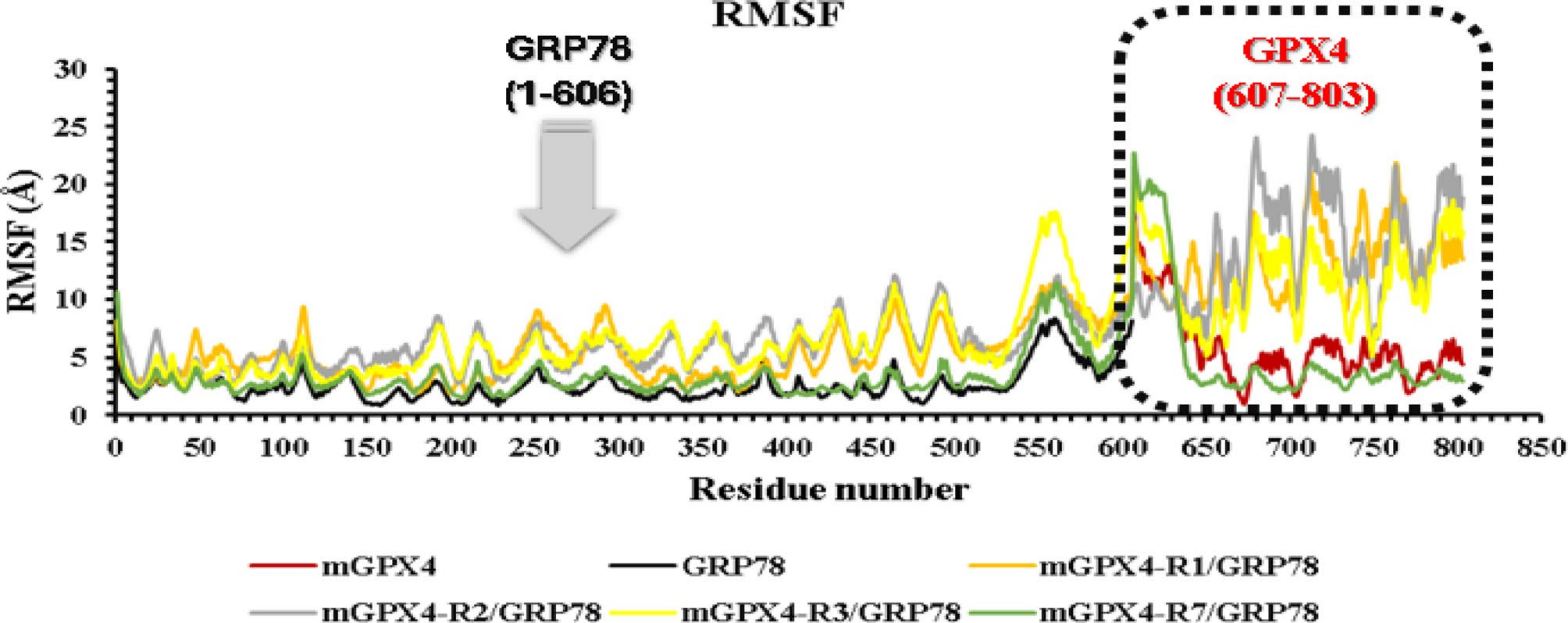
Root-mean-square fluctuation (RMSF) for residues of each protein and the 4 complexes (GRP78-mGPX4). mGPX4 (red), GRP78 (black), complex1(mGPX4-R1/GRP78) (orange), complex2 (mGPX4-R2/GRP78) (gray), complex3 (mGPX4-R3/GRP78) (yellow), and complex4 (mGPX4-R7/GRP78) (green).

Alongside the RMSD assessment, RMSF analysis was carried out to measure residue-level flexibility (Fig. 9). Unbound mGPX4 displayed an average RMSF of 5.79 Å, whereas GRP78 was substantially more rigid at 2.61 Å, reflecting its inherent structural stability. Upon complex formation, GRP78 binding at region VII yielded the lowest RMSF (3.82 Å) of all mGPX4–GRP78 complexes, indicating pronounced restriction of local residue motions. This reduction in flexibility, combined with the moderate RMSD observed for the complex, highlights GRP78’s stabilizing effect at region VII, likely shielding crucial mGPX4 from solvent exposure and proteolytic cleavage.

In contrast, GRP78 binding at regions I, II, and III raises mGPX4’s RMSF to 7.25–7.99 Å, surpassing the flexibility seen in the unbound protein. These larger fluctuations point to increased local mobility, likely driven by conformational shifts or added flexibility in solvent-exposed areas.

**Fig. 10:**
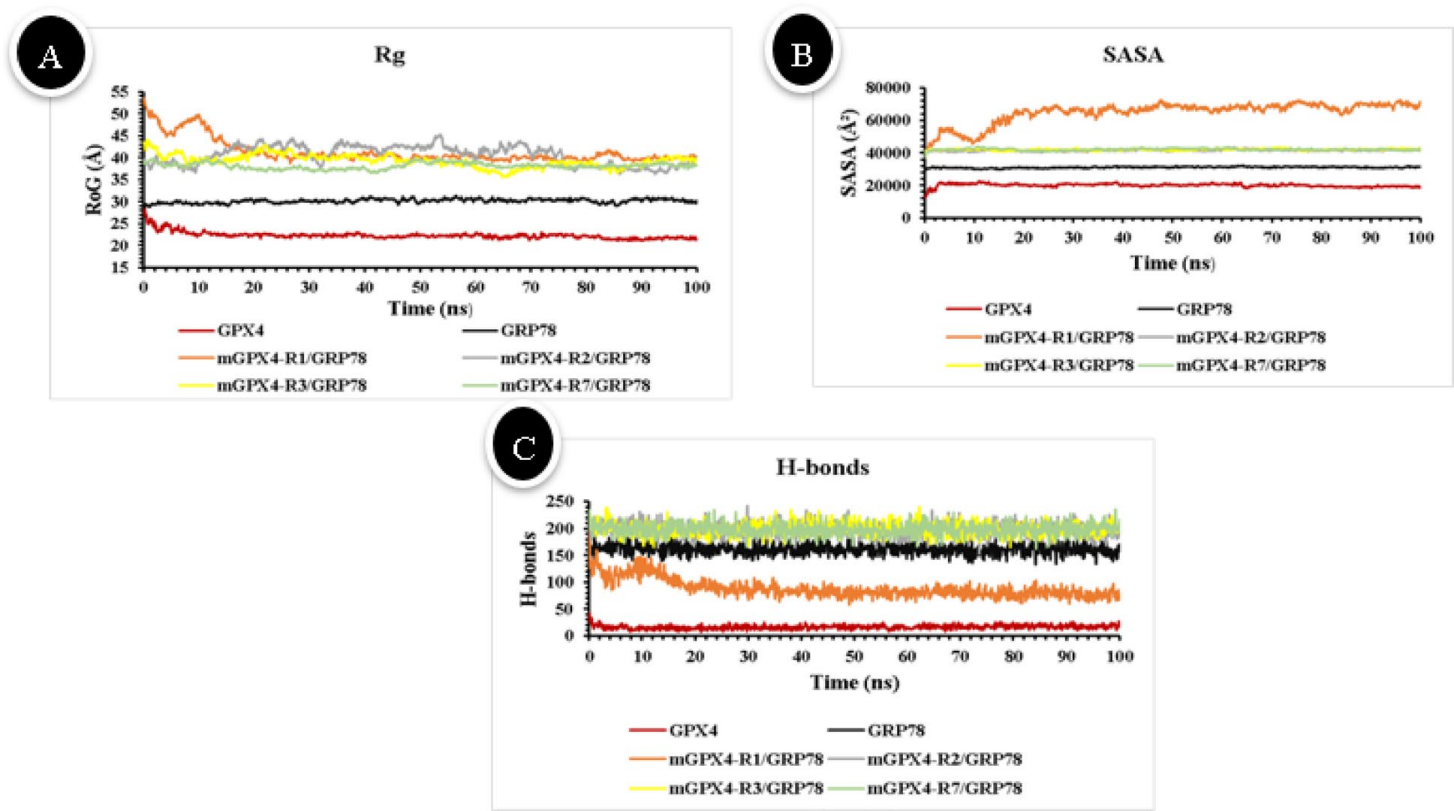
Illustration of (**A**) radius of gyration (Rg, Å), (**B**) solvent-accessible surface area (SASA, Å²), and (C) Hydrogen bonding versus simulation time (nanoseconds) for each protein and the 4 complexes (GRP78-mGPX4). mGPX4 (red), GRP78 (black), complex1(mGPX4-R1/GRP78) (orange), complex2 (mGPX4-R2/GRP78) (gray), complex3 (mGPX4-R3/GRP78) (yellow), and complex4 (mGPX4-R7/GRP78) (green).

Rg quantifies a protein’s compactness and overall structural configuration throughout a simulation. In this study, we compared Rg values to assess how GRP78 binding influences the three-dimensional conformation of mGPX4 (Fig. 10A). Unbound mGPX4 exhibited a compact conformation, with an Rg of 22.30 Å under physiological conditions. In contrast, GRP78 displayed a larger Rg of 30.16 Å, reflecting its chaperone-like architecture and the structural flexibility required for binding to unfolded proteins. When GRP78 is associated with mitochondrial GPX4, Rg of the resulting complex grows substantially, and the extent of this increase is dictated by the binding site. The complexes corresponding to the N-terminal mitochondrial targeting regions I, II, and III exhibit the highest Rg values—41.31 Å, 40.60 Å, and 39.36 Å, respectively.

Such an expansion indicates that GRP78 binding at these sites relaxes the protein’s structure, increasing its flexibility and likely unveiling or shifting the mitochondrial import signals. This behavior aligns with GRP78’s known function in inducing the conformational adjustments needed for protein translocation into organelles^35,36^. Conversely, Complex4 (mGPX4-R7/GRP78) displayed a smaller radius of gyration (38.13 Å) with minimal variation over time, indicating a more compact and stable structure.

SASA analysis was performed to evaluate how GRP78 binding influences the solvent exposure and potential stability of mGPX4. Unbound mGPX4 displayed an average SASA of 20148.06 Å², consistent with a compact conformation. Upon complex formation with GRP78, SASA values increased, reflecting greater solvent accessibility. The mGPX4-R1/GRP78 complex showed the largest average SASA (64851.29 Å²), suggesting that R1 binding induces a more open, flexible, and potentially less stable conformation. In contrast, the mGPX4-R2/GRP78, mGPX4-R3/GRP78, and mGPX4-R7/GRP78 complexes exhibited more moderate SASA increases (41673.33 Å², 41998.95 Å², and 42168.02 Å², respectively) with smaller fluctuations, suggesting these interactions help maintain a relatively compact, stable conformation (see Fig. 10B).

To further evaluate the stabilizing effect of GRP78 on mGPX4, we performed hydrogen-bond analyses over 100-ns simulation trajectories. Unbound mGPX4 displayed a low mean H-bond count (16.45), reflecting a sparse intramolecular network in isolation. In contrast, GRP78 alone formed an average of 162 hydrogen bonds, consistent with its larger size and more complex fold. When GRP78 bound to mGPX4 at regions II, III, and VII, the average number of H-bonds rose sharply to 198, 200, and 199, respectively. These elevated values, coupled with modest errors (± 11–12), indicate stable, persistent interactions throughout the simulations. By comparison, the mGPX4-R1/GRP78 complex averaged just 88 hydrogen bonds with greater variability (± 18), suggesting weaker or transient contacts. Taken together, these results show that GRP78 binding at regions II, III, and VII markedly strengthens mGPX4’s structural integrity via a dense, stable H-bond network, whereas region I engagement yields less favorable stabilization (see Fig. 10C).

We validated the reliability of our docking and MD findings by comparing them with previously reported experimental data and computational studies. Multiple reports have shown that GRP78 directly interacts with GPX4 to preserve its stability, lending support to the GRP78–mGPX4 complex we modeled^37–39^. In addition, GRP78’s preference for binding exposed, hydrophobic, or partially folded regions aligns with the hydrophobic motifs we identified in mGPX4. As a further check on our workflow, we docked the well-characterized GRP78 ligand Pep42 alongside our mGPX4 segments^40–42^. The fact that several mGPX4 regions scored higher in predicted affinity than Pep42 confirms the robustness of our docking protocol and places our calculated GRP78–mGPX4 affinities squarely within a biologically meaningful range. Together, these comparisons validate our computational strategy and underscore the physiological relevance of the GRP78–mGPX4 interaction.

#### Binding free energy results and energetic contributions

**Fig. 11:**
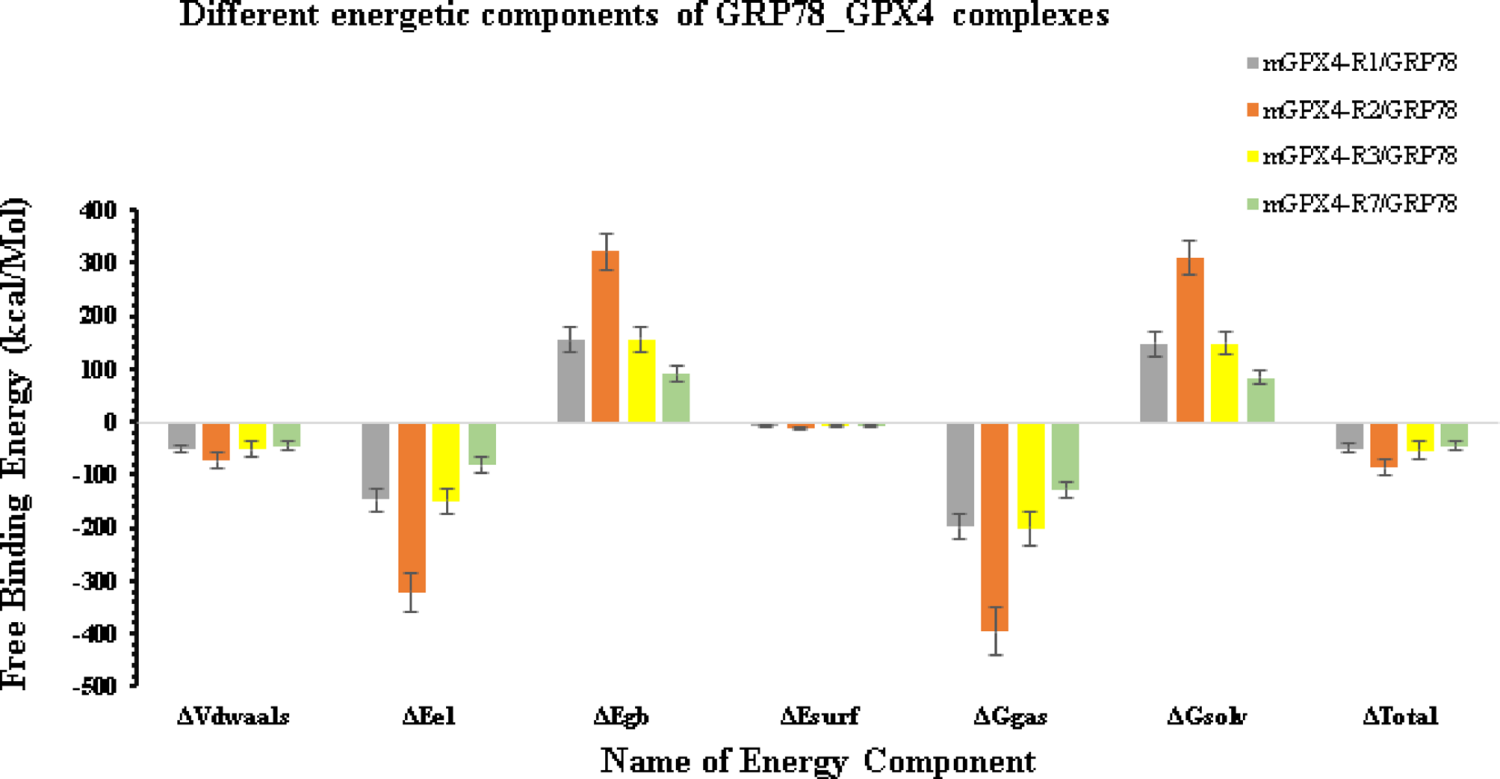
The MM-GBSA calculations for the four complexes (GRP78-mGPX4) after a 100 ns molecular dynamics simulation.

MM-GBSA analysis was carried out on the four GRP78–mGPX4 complexes following 100 ns of molecular dynamics (Fig. 11) to determine their binding free energies and individual energetic contributions. With a total binding free energy of −86.39 kcal/mol, Complex 2 (mGPX4–R2/GRP78) showed the best results, followed by Complex 3 (−53.98 kcal/mol), Complex 1 (−50.10 kcal/mol), and Complex 4 (−45.20 kcal/mol). The exceptionally high affinity of Complex 2 stems mainly from strong electrostatic interactions (ΔEel = − 321.56 kcal/mol) and significant van der Waals forces (ΔVdwaals = − 74.25 kcal/mol). Complexes 1 and 3 also benefit from significant electrostatic and van der Waals contributions, yielding moderately strong binding. A complete list of the energetic contributions of the different complexes is found in the supplementary Table S2.

The per-residue binding energy contributions (all below − 1 kcal/mol) for the GRP78–mGPX4 complexes were calculated and listed in Table 3, underlining each residue’s role in stabilizing the interaction. GPX4 ARG8 (–2.1574 kcal/mol) and GRP78 ARG1488 (–2.6711 kcal/mol) contributed the most to Complex 1 (mGPX4-R1/GRP78). Other significant contributors were GPX4 LEU19 (–2.1335 kcal/mol), LEU15 (–2.1208 kcal/mol), and GRP78 PRO1487 (–2.3269 kcal/mol) and PRO1485 (–2.2037 kcal/mol). Furthermore, the mGPX4-R1 residues ARG8, LEU15, CYS7, LEU14, and CYS16, as well as the SBD residue VAL1432 of GRP78, were essential for maintaining binding stability.

In the mGPX4-R2/GRP78 complex2, the largest binding-energy contributions came from GRP78’s ARG1488 (–2.5213 kcal/mol) and GPX4’s LEU24 (–3.3356 kcal/mol). Additional significant contributors were GRP78 VAL1453 (–2.2505 kcal/mol), GLN1449 (–2.0766 kcal/mol), VAL1490 (–2.0583 kcal/mol), mGPX4-R2 MET28 (–3.1049 kcal/mol), ALA20 (–2.5322 kcal/mol), and LEU19 (–2.2569 kcal/mol). Moreover, GRP78’s substrate-binding domain residue SER1452 and a set of mGPX4-R2 residues (LEU24, MET28, ALA20, LEU19, GLY23, CYS16, PRO22, ALA21, CYS29, ALA18, THR27, and GLY26) were crucial for stabilizing the interaction.

In the mGPX4-R3/GRP78 complex3, GRP78 VAL1490 (–3.3034 kcal/mol) and mGPX4-R3 MET28 (–7.2934 kcal/mol) made the strongest contributions. Other notable residues were GRP78 ARG1488 (–2.2761 kcal/mol), GLN1492 (–2.1708 kcal/mol), and mGPX4-R3 THR27 (–2.5538 kcal/mol). GRP78’s SER1452and VAL1432 and mGPX4-R3 residues MET28, THR27, CYS29, LEU9, ALA25, GLY26, and CYS7 played key roles, underscoring their importance in binding stability.

In Complex 4 (mGPX4-R7/GRP78), the strongest per-residue contributions to binding free energy come from ARG1488 in GRP78 (−4.1821 kcal/mol) and LYS 126 in GPX4 (−3.4808 kcal/mol). Additional significant contributions are observed for Val1453 in GRP78 (−2.8899 kcal/mol), and for LYS58 (−2.4872 kcal/mol) and ALA88 (–2.0104 kcal/mol) in mGPX4-R7. Within the substrate-binding domain of GRP78, residues VAL1429, SER1452, and VAL1432, together with ARG96 in the mGPX4-R7 segment, also play key roles in stabilizing the interface.

**Table 3:** MM/GBSA-calculated per-residue binding free energy contributions for the four complexes.

Complex	GRP78’s residue	Binding energy (kcal/mol)	mGPX4’s residue	Binding energy (kcal/mol)
Complex1 (mGPX4-R1/GRP78)	ARG:1488	−2.6711	ARG:8	−2.1574
	PRO:1487	−2.3269	LEU:19	−2.1335
	PRO:1485	−2.2037	LEU:15	−2.1208
	PRO:1484	−1.9356	CYS:7	−1.9758
	VAL:1432	−1.5341	LEU:14	−1.4585
	VAL:1490	−1.3728	LEU:6	−1.4444
	ILE:1450	−1.3378	ALA:18	−1.3594
	GLY:1489	−1.2008	CYS:16	−1.0004
	VAL:1453	−1.1312		
Complex2 (mGPX4-R2/GRP78)	ARG:1488	−2.5213	LEU:24	−3.3356
	VAL:1453	−2.2505	MET:28	−3.1049
	GLN:1449	−2.0766	ALA:20	−2.5322
	VAL:1490	−2.0583	LEU:19	−2.2569
	LYS:1447	−1.6657	GLY:23	−1.8802
	ILE:1450	−1.6418	CYS:16	−1.7642
	GLU:1217	−1.6038	PRO:22	−1.6142
	SER:1452	−1.5998	ALA:21	−1.3356
	ASN:1219	−1.4720	CYS:29	−1.2577
	VAL:1245	−1.4128	LEU:15	−1.2482
	SER:1448	−1.3268	ALA:18	−1.2334
	THR:1236	−1.1829	THR:27	−1.0958
	GLY:1489	−1.0770	GLY:26	−1.0547
			LEU:14	−1.0401
Complex3 (mGPX4-R3/GRP78)	VAL:1490	−3.3034	MET:28	−7.2934
	ARG:1488	−2.2761	THR:27	−2.5538
	GLN:1492	−2.1708	CYS:29	−1.7843
	GLY:1513	−1.9200	ALA:30	−1.4116
	THR:1514	−1.7794	LEU:9	−1.3884
	GLY:1489	−1.7344	ARG:32	−1.2951
	PRO:1487	−1.7016	LEU:6	−1.2276
	VAL:1453	−1.4342	ALA:25	−1.0780
	VAL:1432	−1.3108	GLY:26	−1.0547
	PRO:1491	−1.1476	CYS:7	−1.0456
	SER:1452	−1.0611		
Complex4 (mGPX4-R7/GRP78)	ARG:1488	−4.1821	LYS:126	−3.4808
	VAL:1453	−2.8899	LYS:58	−2.4872
	VAL:1429	−1.8733	ALA:88	−2.0104
	SER:1452	−1.7196	ALA:91	−1.6003
	GLY:1430	−1.7193	HSD:87	−1.5806
	VAL:1432	−1.7004	ARG:96	−1.3599
	GLY:1454	−1.5952		
	PRO:1487	−1.2976		
	PRO:1491	−1.2825		
	VAL:1490	−1.1060		
	GLY:1455	−1.0343		
	THR:1456	−1.0289		

**Fig. 12:**
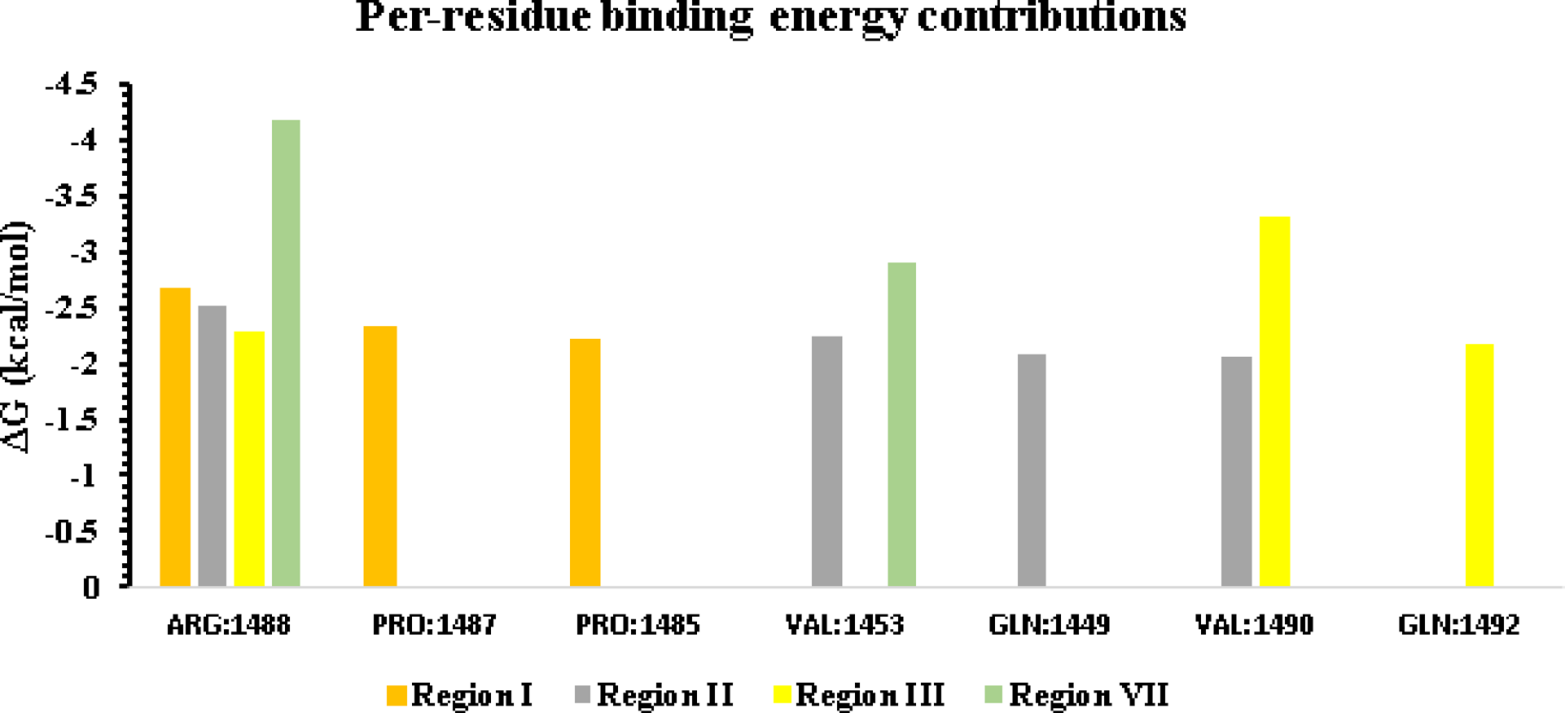
The contributions of important residues (with **Δ**G < − 2 kcal/mol) at the GRP78 SBDβ domain’s active region.

Figure 12 illustrates the energetic contributions (ΔG < − 2 kcal/mol) of key residues located within the active regions of the GRP78 SBDβ domain. ARG1488 consistently showed strong binding across all four subregions, with its greatest effect in Region VII (ΔG = − 4.1821 kcal/mol), underscoring its central role in the interaction. The ΔG values of PRO1487 and PRO1485 were − 2.3269 kcal/mol and − 2.2037 kcal/mol, respectively, and they were only active in Region I. In Region II, VAL1453, GLN1449, and VAL1490 each contributed substantially (ΔG ranging from − 2.0583 to − 2.2505 kcal/mol). Region III was dominated by VAL1490 (ΔG = − 3.3034 kcal/mol), alongside a supporting role from GLN1492 (ΔG = − 2.1708 kcal/mol). VAL1453 also exhibited a notable interaction in Region VII (ΔG = − 2.8899 kcal/mol). Together, these results highlight ARG1488, VAL1490, and VAL1453 as key residues that stabilize binding across different mGPX4-GRP78 SBDβ complexes.

Complex 2 (mGPX4-R2/GRP78) has the most advantageous total binding free energy (–86.39 kcal/mol), according to the MM/GBSA analysis. This is mostly due to van der Waals (–74.25 kcal/mol) and electrostatic (–321.56 kcal/mol) interactions. Favourable non-polar solvation (ΔEsurf = − 12.28 kcal/mol) somewhat offsets the significant polar solvation penalty (+ 321.70 kcal/mol) associated with this strong binding, making R2 an energetic hotspot for GRP78 engagement.

In contrast, Complex 4 has a less favourable total binding free energy (–45.20 kcal/mol), but it has a more compact and structurally stable interface with balanced van der Waals (–45.36 kcal/mol) and electrostatic (–82.85 kcal/mol) contributions and a comparatively low solvation penalty. Comparative analysis of Complexes 1 and 3 indicates intermediate or lower binding affinities (− 50.10 and − 53.98 kcal/mol, respectively, reflecting weaker electrostatic and non-polar interactions. Together, these results suggest that while the region II region yields the strongest energetic driving force for GRP78 binding, the region VII region forms a tighter, potentially more stable complex, making both sites functionally important for the GRP78–mGPX4 interaction.

The computational results are consistent with the findings of Song et al., who found that gefitinib-resistant MDA-MB-231/Gef and HS578T/Gef TNBC cell lines had higher levels of GPX4, indicating a role for GPX4 in oxidative-stress adaptation and drug resistance^43^. Direct GPX4 inhibitors have shown promising outcomes. In TNBC models, parthenolide derivative DMOCPTL interacts with GPX4, increases its ubiquitination, and triggers ferroptosis and apoptosis. DMOCPTL considerably reduced tumour growth and increased lifespan in vivo while posing little harm^44^. All of the research points to GPX4 as a possible target for treatment in TNBC. The oncogenic significance of GRP78 in TNBC is supported by experimental findings. In MDA-MB-231 and BT549 cells, GRP78 knockdown significantly reduced colony formation and proliferation, underscoring its involvement in TNBC’s aggressiveness^45^. GRP78 is not only overexpressed in TNBC cells but also exists as distinct cell-surface subpopulations that correlate with metastatic behavior. Specifically, treatment with chemotherapy agents such as doxorubicin induces an increase in the GRP78-positive (GRP78⁺) cell population in TNBC lines (e.g., MDA-MB-468 and MDA-MB-231), which display reduced invasiveness and migratory capacity compared to GRP78-negative (GRP78⁻) cells^46^.

Although GRP78 is classically described as an ER-resident chaperone, multiple reports have documented its relocation to mitochondria under stress. During the unfolded protein response, GRP78 traffics into the mitochondrial inner membrane, intermembrane space, and matrix^1,47^. In cancer cells, Teng et al. (2015) found GRP78 bound to the mitochondrial protein ATAD3A, where it helps stabilize membrane‐associated proteins^48^. Under ER stress in prostate cancer, GRP78 also mediates the retrotranslocation of clusterin into mitochondria^49^. Together, these findings make it plausible that GRP78 could co‐localize with mitochondrial GPX4 when cells are stressed.

Independent studies further implicate mitochondrial GRP78 in TNBC. Immunohistochemical profiling of TNBC specimens reveals significantly elevated GRP78 levels across several patient cohorts^50^. A TNBC-specific mechanistic study demonstrated that miR-6126 directly targets GRP78 to modulate mitochondrial dynamics, metabolic reprogramming, and the Warburg effect^51^. Likewise, GPX4 is critical for TNBC survival, ferroptosis resistance, and drug tolerance: gefitinib-resistant TNBC lines express higher GPX4 levels, and GPX4 knockdown restores sensitivity to treatment^43^. Despite these converging roles in TNBC mitochondria, no study has yet co-isolated GPX4 with mitochondrial GRP78, leaving their potential interaction a compelling but experimentally unverified hypothesis.

From a mechanistic perspective, our computational model of the GRP78–mGPX4 interaction dovetails neatly with published experimental data. Multiple studies have shown that GRP78 (HSPA5) binds directly to GPX4, shielding it from degradation and thus blocking ferroptosis under stress. In pancreatic cancer models, HSPA5 associates with GPX4 to prevent its proteolytic loss, curbing lipid peroxidation and ferroptotic cell death^10^. In colorectal cancer, HSPA5 slows GPX4 depletion during erastin-induced ferroptosis, delaying its breakdown and promoting cell survival^38,52^. And in glioma, reducing HSPA5 levels lowers GPX4 expression and activity, rendering cells more susceptible to dihydroartemisinin-triggered ferroptosis—further evidence of its chaperone-like role^37^. Taken together, these findings strongly support our in silico prediction that GRP78 stabilizes mGPX4 in TNBC, contributes to ferroptosis resistance, and represents a viable target for therapeutic disruption.

Our computational results highlight a new therapeutic strategy: disrupting the GRP78–GPX4 interaction to destabilize GPX4, weaken its protective function, and thereby sensitize TNBC cells to ferroptosis-inducing agents. Because TNBC lacks specific targeted therapies and frequently acquires resistance to standard treatments, this approach could serve as a valuable complementary or adjuvant option. Small molecules, peptides, or antibodies that block GRP78’s stabilization of GPX4 have the potential to amplify the effectiveness of ferroptosis-based therapies, bypass resistance mechanisms, and ultimately improve clinical outcomes in TNBC.

## Conclusion

GRP78 maintains ER homeostasis in healthy cells by governing the unfolded protein response: it helps refold misfolded proteins or targets them for degradation. In TNBC, GRP78 also suppresses ferroptosis. The selenoprotein GPX4—a key ferroptosis regulator—uses glutathione to prevent lipid peroxidation. Their interaction promotes tumor cell survival, proliferation, and metastasis, and because this complex is often overexpressed in cancer, it represents a promising therapeutic target. Interrupting the binding between GPX4 and GRP78’s substrate-binding domain β (SBDβ) is expected to lower ferroptosis resistance and boost anticancer efficacy. In our study, we pinpoint novel contact sites between active GRP78 SBDβ residues and mitochondrial GPX4. Of the regions analyzed, region II delivers the highest binding energy, while region VII forms the most compact, potentially stable complex, making both critical for the GRP78–mGPX4 interaction. These findings lay the groundwork for designing inhibitors that disrupt this complex, to halt tumor growth, overcome resistance, and undermine cancer cell survival. While our computational analyses shed light on the GPX4–GRP78 interaction, these findings need to be confirmed by additional experimental validation in TNBC models in order to assess their therapeutic significance.

## Supplementary Information

Below is the link to the electronic supplementary material.

Supplementary Material 1

## Data Availability

All data generated or analyzed during this study are included in this published article.
