# Supplementary material for: Computational characterization of GRP78 binding sites on mitochondrial GPX4: implications for targeting ferroptosis in triple-negative breast cancer
Source: Sci Rep. 2026 Jan 10;16:1330. doi: 10.1038/s41598-025-33108-1 (PMC12796417; doi:10.1038/s41598-025-33108-1)
Supplement: Supplementary file 1 — Supplementary Material 1 [file 41598_2025_33108_MOESM1_ESM.docx]

*Table S1. The docking score and binding affinity (∆G) for interactions between specific regions of mGPX4 and GRP78 were analyzed using the HADDOCK web server. The top-ranked conformation, highlighted in bold, demonstrated the highest binding affinity (most negative value) with the GRP78 SBDβ compared to other clusters. GRP78 with Pep42 is used as a reference model*

| **Complex** | **Cluster** | **HADDOCK**  **Score** | **Conformations** | **Binding affinity**  **ΔG (kcal/mol)** |
| --- | --- | --- | --- | --- |
| **Pep42/GRP78** | Cluster (2) | -46.4 ± 1.8 | **Nr (1)** | **-7.1** |
|  |  |  | Nr (2) | -6.7 |
|  |  |  | Nr (3) | -6.7 |
|  |  |  | Nr (4) | -6.9 |
| **Complex1**  **(mGPX4-R1/GRP78)** | Cluster (2) | -54.1 ± 2.6 | Nr (1) | -7.7 |
|  |  |  | Nr (2) | -7.6 |
|  |  |  | Nr (3) | -7.8 |
|  |  |  | **Nr (4)** | **-8.0** |
| **Complex2**  **(mGPX4-R2/GRP78)** | Cluster (1) | -72.0 ± 5.4 | Nr (1) | -10.4 |
|  |  |  | **Nr (2)** | **-11.0** |
|  |  |  | Nr (3) | -10.2 |
|  |  |  | Nr (4) | -9.9 |
| **Complex3**  **(mGPX4-R3/GRP78)** | Cluster( 5) | -55.5 ± 3.0 | Nr (1) | -10.0 |
|  |  |  | Nr (2) | -10.4 |
|  |  |  | Nr (3) | -10.3 |
|  |  |  | **Nr (4)** | **-11.3** |
| **Complex4**  **(mGPX4-R7/GRP78)** | Cluster (7) | -68.3 ± 4.6 | Nr (1) | -7.7 |
|  |  |  | Nr (2) | -7.5 |
|  |  |  | **Nr (3)** | **-8.4** |
|  |  |  | Nr (4) | -7.3 |

***Table S2.*** *The MM-GBSA calculations for the four complexes (GRP78-mGPX4) after 100 ns molecular dynamics simulation: Van der Waals energy (ΔVdwaals), electrostatic energy (ΔEel), generalized Born solvation energy (ΔEgb), solvent-accessible surface area energy (ΔEsurf), gas-phase interaction energy (Δggas), solvation free energy (ΔGsolv), and total free energy (ΔTotal).*

| **Energy Component (kcal/mol)** | **Complex1**  **(mGPX4-R1/GRP78)** | **Complex2**  **(mGPX4-R2/GRP78)** | **Complex3**  **(mGPX4-R3/GRP78)** | **Complex4**  **(mGPX4-R7/GRP78)** |
| --- | --- | --- | --- | --- |
| ΔVdwaals | -50.56 | -74.25 | -52.58 | -45.36 |
| ΔEel | -147.19 | -321.56 | -149.13 | -82.85 |
| ΔEgb | 154.38 | 321.70 | 155 | 89.84 |
| ΔEsurf | -6.74 | -12.28 | -7.28 | -6.83 |
| ΔGgas | -197.74 | -395.81 | -201.7 | -128.21 |
| ΔGsolv | 147.64 | 309.43 | 147.72 | 83.01 |
| ΔTotal | -50.10 | -86.39 | -53.98 | -45.20 |
